# Prediction of Poor Prognosis in Breast Cancer Patients Based on MicroRNA-21 Expression: A Meta-Analysis

**DOI:** 10.1371/journal.pone.0118647

**Published:** 2015-02-23

**Authors:** Yanyan Wang, Yujie Zhang, Chi Pan, Feixia Ma, Suzhan Zhang

**Affiliations:** 1 Department of Surgical Oncology, Second Affiliated Hospital, Medicine School of Zhejiang University, Hangzhou, China; 2 Department of Orthopaedic Surgery, Second Affiliated Hospital, Medicine School of Zhejiang University, Hangzhou, China; 3 Cancer Institute, Second Affiliated Hospital, Medicine School of Zhejiang University, Hangzhou, China; University of Tennessee Health Science Center, UNITED STATES

## Abstract

**Background:**

MicroRNA-21 (miRNA-21 or miR-21) may act as a prognostic biomarker of cancer. However, the available evidence is controversial. Therefore, the present meta-analysis summarizes this evidence and evaluates the prognostic role of this gene in breast cancer.

**Methods:**

The meta-analysis was conducted by searching the databases of PubMed, EMBASE, Web of Science and Chinese database-China National Knowledge Infrastructure (CNKI). Data were extracted from studies that investigated the association between miR-21 expression and survival outcomes in breast cancer patients. With respect to survival outcomes, the pooled hazard ratios (HRs) of miR-21 were calculated given a 95% confidence interval (CI).

**Results:**

Our meta-analysis identified a total of 10 studies involving 1,439 cases. Further investigation demonstrated that a high miR-21 expression can predict poor overall survival (OS) (HR = 2.57, 95% CI: 1.37—4.81, *P* = 0.003) and shortened disease-free/recurrence-free survival (DFS/RFS) (HR = 1.45, 95% CI: 1.16—1.82, *P* = 0.001) in breast cancer patients. Moreover, high miR-21 expression was significantly correlated with lowered OS in the Asian group (HR = 5.07, 95% CI: 2.89—8.92, *P* < 0.001), but not in the Caucasian cohort (HR = 1.44, 95% CI: 0.99—2.10, *P* = 0.058). Furthermore, odds ratios (ORs) showed that up-regulated miR-21 levels were associated with multiple clinical characteristics.

**Conclusion:**

Our results indicated that miR-21 can predict unfavorable prognoses in breast cancer patients, especially in Asians.

## Introduction

Breast cancer is the most frequently diagnosed malignancy and is the leading cause of cancer death among females. It accounts for 23% of all cancer cases and 14% of cancer deaths worldwide [1]. Approximately 232,670 women in the United States are estimated to be diagnosed with invasive breast cancer in 2014, and 40,000 women will die from it [2]. At present, mortality rates are declining in several Western countries because of the increased implementation of mammographic screening and adjuvant systemic therapies for newly diagnosed cases. However, the long-term survival rate and prognosis of advanced-stage patients remain poor, especially in developing countries [2,3]. Currently, both tissue- and serum-based tumor biomarkers are widely used to screen early-stage breast cancer and to predict either its progression or recurrence in advance. These biomarkers include human epidermal growth factor 2 receptor (Her2), estrogen receptor (ER), progesterone receptor (PR), and p53 [4]. Breast cancer is quite complex and heterogeneous in its development, progress, and response to treatment; therefore, researchers are encouraged to identify novel biomarkers for the optimization of breast cancer management.

MiRNAs are a class of 18–25 nucleotide, non-coding RNAs that are significant in the regulation of post-transcriptional gene expression [5]. Studies have shown that miRNAs are involved in virtually all biological processes, including cell proliferation, differentiation, and apoptosis [5,6]. Moreover, miRNAs act as critical regulators of tumorigenesis and are believed to be involved in tumor development and progression [7]. Calin *et al*. were the first to provide direct evidence of regarding the function of miRNA in human cancer. They reported that aberrant expressions of both miR-15 and miR-16 are related to chronic lymphocytic leukemia [8]. Many subsequent studies identified several miRNAs that are correlated with various human cancers [9,10]. In addition, clinical and quantitative studies determined that the expression levels of some miRNAs are associated with either the stages of diseases or with cancer survival outcomes [11,12]; thus, miRNAs may serve as potential diagnostic or prognostic biomarkers of cancer.

MiR-21 acts as an oncogene and is among the most frequently observed cancer-related miRNAs. It is consistently dysregulated in many types of cancer [13] and is a key factor in tumorigenesis and tumor suppression because it targets tumor suppressor genes such as tropomyosin 1, programmed cell death 4, and phosphatase and tensin homolog [14–17]. Recent accumulated evidence supports the concept of miR-21 as a potential diagnostic or prognostic biomarker in various types of cancer, such as colon cancer, pancreatic cancer, and lung cancer [18–20]. Nonetheless, some of these studies fail to confirm the association between miR-21 and cancer survival outcomes [21,22]; as a result, their results remain controversial. Moreover, the prognostic value of miR-21 varies in different clinical studies with respect to breast cancer [23,24]. Therefore, we conduct this meta-analysis to determine whether miR-21 can predict poor survival rates in breast cancer patients.

## Materials and Methods

### Search strategy and study selection

**Fig 1:**
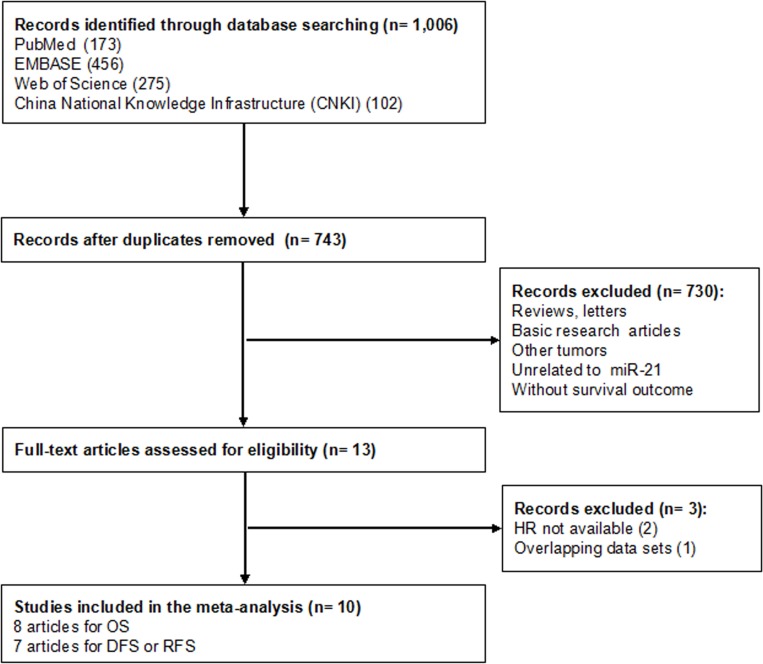
Flow diagram of the study selection process.

We searched the international databases (PubMed, EMBASE and Web of Science) and Chinese database-China National Knowledge Infrastructure (CNKI) carefully for relevant articles (last update: 24 November 2014). The following key words or text words were used: “breast or mammary”, “cancer or carcinoma”, “tumor or tumour”, and “microRNA-21, miR-21, or miRNA-21”. Studies were considered eligible if they met the following criteria: (i) They diagnosed breast cancer based on histopathological confirmation; (ii) they measured the miR-21 expression in either tumor tissue or serum; and (iii) they investigated the association between the expression level of miR-21 and survival outcomes. Articles were excluded based on any of the following criteria: (i) Reviews, comments, conference abstracts, letters, or basic research articles; (ii) neither English nor Chinese articles; and (iii) those that lacked key information, such as HR, 95% CI, and *P* value, or useful data for the calculation developed by Parmar, Williamson, and Tierney [25–27]. When multiple publications of a study were identified, we selected the most detailed version for meta-analysis. Two reviewers (YYW and YJZ) identified the qualified studies independently in accordance to the eligibility criteria. Discrepancies were adjudicated by a third reviewer (FXM) until a consensus was reached. A flow diagram of the study selection process is presented in Fig. 1.

### Quality assessment

Based on a critical review checklist of the Dutch Cochrane Centre proposed by MOOSE, we systematically assessed the quality of all the studies included [28]. The key points of a qualified study include: (i) Detailed information regarding the study population and country of origin; (ii) a clear description of the study design; (iii) a clear description of outcome assessment; (iv) an adequate description of miR-21 measurement; (v) a clear description of the cut-off value of miR-21; and (vi) a sufficient follow-up period. We excluded the studies that did not meet all six of these points.

### Data extraction

Two reviewers (YYW and YJZ) extracted the required information from all eligible studies independently. The extracted data included the following: (i) Publication information (name of the author cited first and the publication year); (ii) patients characteristics (age, ethnicity, sample site, stage of disease, histological grade, lymphoid node status, subtypes, Her2/ER/PR status, and follow-up); (iii) miR-21 measurement and cut-off value; and (iv) the HRs of the elevated miR-21 for overall survival (OS), disease-free survival (DFS), recurrence-free survival (RFS), as well as their 95% CIs and *P* values. If miR-21 expression was divided into several categories in a particular study, we combined the corresponding HR estimates using the method proposed by Hamling *et al*. [29]. If HRs were not provided, we calculated these values based on the total numbers of observed deaths or cancer recurrences and the numbers of samples in each group. If the Kaplan–Meier curves alone were available, we extracted data from the graphical survival plots to estimate the HRs [27]. We also e-mailed the authors of the selected articles to request for additional information and for copies of the original data required for the meta-analysis.

### Statistical analysis

The heterogeneity of the combined HRs was evaluated with Cochran’s Q test and the Higgins I-squared statistic. The HRs were considered statistically heterogeneous if they displayed *P* < 0.05 and/or I^2^ > 50% [30]. Significant heterogeneities among the studies were resolved with the random-effects model (DerSimonian-Laird method) [31]. Otherwise, the fixed-effects model (Mantel-Haenszel method) was applied [32]. Subgroup analysis was conducted to determine the source of existing heterogeneity. HR values > 1 indicated significant associations with poor prognosis. We also examined the correlation between miR-21 expression and the clinical variables in breast cancer through odds ratio (OR) [33]. Publication bias was analyzed using the funnel plot in combination with Egger's test (*P* > 0.05 suggested a lack of publication bias). Finally, the influence of a single study on overall HR was assessed for sensitivity analysis. All analyses were conducted with “Stata: Data Analysis and Statistical Software” V12.0 (Stata Corporation, College Station, TX, U.S.).

## Results

### Summary of the included studies

As shown in Fig. 1, a total of 1,006 published records were initially retrieved in the databases of PubMed, EMBASE, Web of Science and CNKI, and 263 of them were excluded due to duplication. According to the exclusion criteria, 730 studies were further removed based on manual screening of the titles and abstracts. Of the remaining 13 candidate articles, two articles did not provide the essential data for the extraction of HRs and 95% CI [34,35], one study reported duplicate data that had been published previously [36]. A final total of 10 studies, 8 for OS, 7 for DFS/RFS, respectively, were considered in the meta-analysis [23,24,37–44].

The main characteristics and results of the eligible studies are summarized in Table 1 and Table 2. These studies investigated a total of 1,439 cases from China, Italy, Korea, Japan, Greece, the United States, and Germany. The patients were classified as either Asian or Caucasian according to their ethnic background. Radojicic *et al*. and Dong *et al*. only selected triple-negative breast cancer cases [24,42], Müller *et al*. enrolled Her2+ breast cancer patients [44], whereas other studies included mixed type of breast cancer patients. MiR-21 expression was detected by quantitative real-time polymerase chain reaction assay in all studies with 8 in cancerous tissue, 1 in bone marrow and 1 in serum. Furthermore, the cut-off values of miR-21 varied in each study. Median and mean values were extracted from eight studies, and 5.84-fold or 1.5-fold values were considered in the remaining studies. Five of these studies focused on both OS and DFS [24,37,39,40,43]; three studies mainly emphasized OS [23,41,44]; and two investigated DFS or RFS [38,42]. Notably, Müller *et al*. examined the serum concentrations of circulating miR-21 in breast cancer patients before and after therapy [44], thus we got 2 HR estimates for OS in one study.

**Table 1:** Main characteristics of enrolled studies.

Author's name	Year	Country	Ethnicity	N	Stage	Type	Sample	Follow-up, months	HR	Results
Yan	2008	China	Asian	113	I-III	Mixed	FFPE	66.2(10.4–81)	Reported	OS
Qian	2009	Italy	Caucasian	344	I-IV	Mixed	Frozen tissue	86.2(8–108)	Reported	OS/DFS
Sun	2009	China	Asian	57	I-III	Mixed	FFPE	56.3(6–209.8)	Reported	DFS
Lee	2011	Korea	Asian	109	I-III	Mixed	FFPE	100	Reported	OS/DFS
OTA	2011	Japan	Asian	291	I-IV	Mixed	Bone marrow	61	Reported	OS/DFS
Radojicic	2011	Greece	Caucasian	49	I-IV	Mixed	FFPE	120	AP/SC	OS/DFS
Walter	2011	USA	Caucasian	25	NM	TNBC	FFPE	median 35.5	SC	OS
Dong	2014	China	Asian	86	I-IV	TNBC	Frozen tissue	95	Reported	RFS^*^
Markou	2014	Greece	Caucasian	112	Early stage	Mixed	FFPE	148.8	Reported	OS/DFS
Müller-b	2014	Germany	Caucasian	127	I-IV	Her2+	Serum	62.15(5.56–66.28)	SC	OS
Müller-a	2014	Germany	Caucasian	126	I-IV	Her2+	Serum	62.15(5.56–66.28)	SC	OS

Abbreviations: FFPE, formalin-fixed paraffin-embedded; AP, author provided; SC, survival curve; NM, not mentioned; OS, overall survival; DFS, disease-free survival; RFS, recurrence-free survival; TNBC, triple-negative breast cancer; Müller-b, results before therapy; Müller-a, results after therapy.

*The extracted OS data is obviously not consistent with the survival curve, so only provided RFS is used for analysis.

**Table 2:** Main results of enrolled studies.

Author’s name	Method	Cut-off	Survival analysis	Conclusion	HR(95% CI) for OS	*P* value	HR(95% CI) for DFS/RFS	*P* value
Yan	qRT-PCR	Mean: 1.741	M	Positive	4.13(1.80–9.50)	0.001		
Qian	qRT-PCR	Median	M	Negative	0.99(0.56–1.73)	NM	1.15(0.69–1.93)	NM
Sun	qRT-PCR	Mean:5.04	U	Negative			1.81(0.96–3.41)	0.066
Lee	qRT-PCR	Mean: 5.92	M	Positive	14.21(1.34–15.10)	0.028	0.88(0.09–8.41)	0.862
OTA	qRT-PCR	5.84	M	Positive	3.40(1.26–9.18)	0.035	1.04(0.71–1.48)	0.853
Radojicic	qRT-PCR	Median: 0.74	KM	Negative	0.85(0.09–8.29)	0.337	2.49(0.72–8.58)	0.379
Walter	qRT-PCR	Median:5	KM	Negative	0.49(0.06–3.71)	0.99		
Dong	qRT-PCR	1.5	U	Positive			2.32(1.24–4.12)	0.033
Markou	qRT-PCR	Median	U	Positive	1.48(0.73–2.98)	0.274	2.49(1.30–4.80)	0.006
Müller-b	qRT-PCR	Median	KM	Positive	5.24(1.58–17.35)	0.009		
Müller-a	qRT-PCR	Median	KM	Positive	3.40(1.01–11.38)	0.037		

Abbreviations: qRT-PCR, quantitative real-time PCR; M, multivariate analysis; U, Univariate analysis; KM, Kaplan–Meier analysis; NM, not mentioned; OS, overall survival; DFS, disease-free survival; RFS, recurrence-free survival; Müller-b, results before therapy; Müller-a, results after therapy.

### Correlation between miR-21 expression and survival outcome

**Fig 2:**
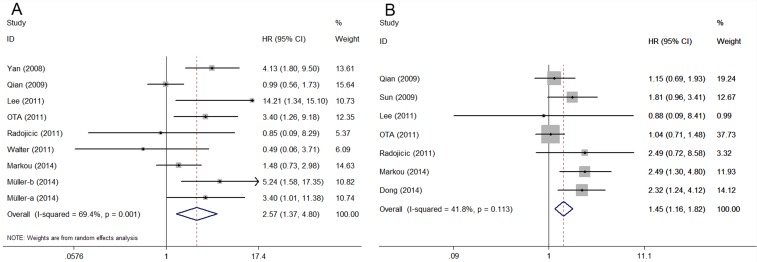
Forest plots of the relationship between elevated miR-21 level and OS (A) and DFS/RFS (B).

Nine OS-related data displayed heterogeneity (I^2^ = 69.4%, *P* = 0.001). Hence, we applied a random model to calculate the pooled HR and its corresponding 95% CI. A high miR-21 expression was significantly associated with poor OS, unlike low miR-21 expression (HR = 2.57, 95% CI: 1.37–4.81, *P =* 0.003) (Fig. 2A). To reduce the influence of heterogeneity, we conducted subgroup analysis according to ethnicity, cut-off value and types of breast cancer, respectively. In the ethnicity subgroup analysis, heterogeneity was considerably dissolved between the Asian (I^2^ = 45%, *P* = 0.162) and Caucasian groups (I^2^ = 46.6%, *P =* 0.096). Moreover, elevated miR-21 reduced the OS of Asian cancer patients (HR = 5.07, 95% CI: 2.89–8.92, *P <* 0.001), but not that of Caucasian ones (HR = 1.44, 95% CI: 0.99–2.10, *P* = 0.058). In the cut-off subgroup analysis, mean cut-off value was significantly associated with OS (HR = 7.06, 95% CI: 2.13–23.45, *P* = 0.001) under a random-effects model because the considerable heterogeneity among the pooled studies (I^2^ = 63.1%, *P* = 0.1). OS was not significantly lowered in the median cut-off group. In the type subgroup analysis, over-expression of miR-21 was predictive of worse OS (HR = 2.41, 95% CI: 1.09–5.32, *P* = 0.029) in mixed type of breast cancer patients by random model with prominent heterogeneity among studies (I^2^ = 77.8%, *P <* 0.001). Similar result was found in Her2+ group but not triple-negative group (Table 3).

**Table 3:** Subgroup analyses of pooled hazard ratios for overall survival with elevated miR-21 expression.

Subgroup analysis	Data sets (number)	Number of patients	Model	HR(95% CI)	*P* value	Heterogeneity (I^2^, P-value)
All	9	1,296	Random	2.57(1.37–4.81)	0.003	69.4%, 0.001
Ethnicity						
Asian	3	513	Fixed	5.07(2.89–8.92)	<0.001	45%, 0.162
Caucasian	6	783	Fixed	1.44(0.99–2.10)	0.058	46.6%, 0.096
Cut-off						
Mean	2	222	Random	7.06(2.13–23.45)	0.001	63.1%, 0.1
Median	6	783	Fixed	1.44(0.99–2.10)	0.058	46.6%, 0.096
Type						
Mixed	6	994	Random	2.41(1.09–5.32)	0.029	77.8%, <0.001
Her2+	2	253	Fixed	4.227(1.81–9.90)	0.001	0%, 0.618
Triple-negative	1	49		0.85(0.09–8.29)	0.337	

A fixed model was used to examine the seven studies that evaluated DFS/RFS given the absence of heterogeneity among the studies (I^2^ = 41.8%, *P* = 0.113). Similarly, miR-21 over-expression significantly predicted poor DFS/RFS in breast cancer (HR = 1.45, 95% CI: 1.16–1.82, *P* = 0.001) (Fig. 2B).

### Correlation between miR-21 expression and clinicopathologic parameters

Based on the ORs derived from each available study, we also evaluated the correlation between miR-21 expression and some clinical characteristics, including TNM stage, lymph node metastasis, histological grade, Her2, ER, and PR status (Table 4). The association between miR-21 expression level and TNM stage was statistically significant (OR = 3.41, 95% CI: 1.92–6.05, *P <* 0.001). This expression level was also similarly correlated with histological grade (OR = 3.02, 95% CI: 1.95–4.70, *P <* 0.001. However, it was not significantly linked to lymph node metastasis (*P =* 0.337). Her2 status was significantly associated with high miR-21 levels (OR = 3.34, 95% CI: 1.97–5.67, *P <* 0.001) and ER status was negatively related to miR-21 expression (OR = 0.53, 95% CI: 0.35–0.80, *P* = 0.002), as well as PR status (OR = 0.49, 95% CI: 0.32–0.74, *P* = 0.001). Detailed information for ORs calculation was summarized in Supporting Information S1 Table.

**Table 4:** Meta-analyses of miR-21 Expression Classified by clinicopathologic parameters.

Variables	Number of studies	Number of patients	Model	OR(95% CI)	*P* value	Heterogeneity(I^2^, P-value)
TNM stage (III/IV vs. I/II)	3 [23,39,42]	294	Fixed	3.41(1.92–6.05)	<0.001	0.0%, 0.469
Lymph node metastasis (positive vs. negative)	5 [23,39–42]	608	Random	1.54(0.64–3.73)	0.337	78.2%, 0.001
Histological grad (III vs. I/II)	4 [39–42]	497	Fixed	3.02(1.95–4.70)	<0.001	0.0%, 0.580
Her2 status (positive vs. negative)	3 [23,39,40]	391	Fixed	3.34(1.97–5.67)	<0.001	13.6%, 0.314
ER status (positive vs. negative)	4 [23,39–41]	529	Fixed	0.53(0.35–0.80)	0.002	48.0%, 0.123
PR status (positive vs. negative)	4 [23,39–41]	524	Fixed	0.49(0.32–0.74)	0.001	0.0%, 0.464

### Publication bias and sensitivity analysis

**Fig 3:**
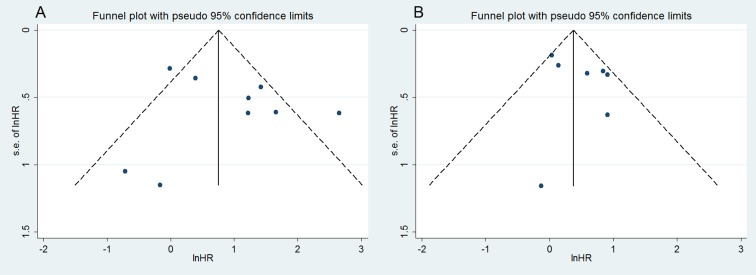
Funnel plot for publication bias analysis: (A) OS; (B) DFS/RFS.

**Fig 4:**
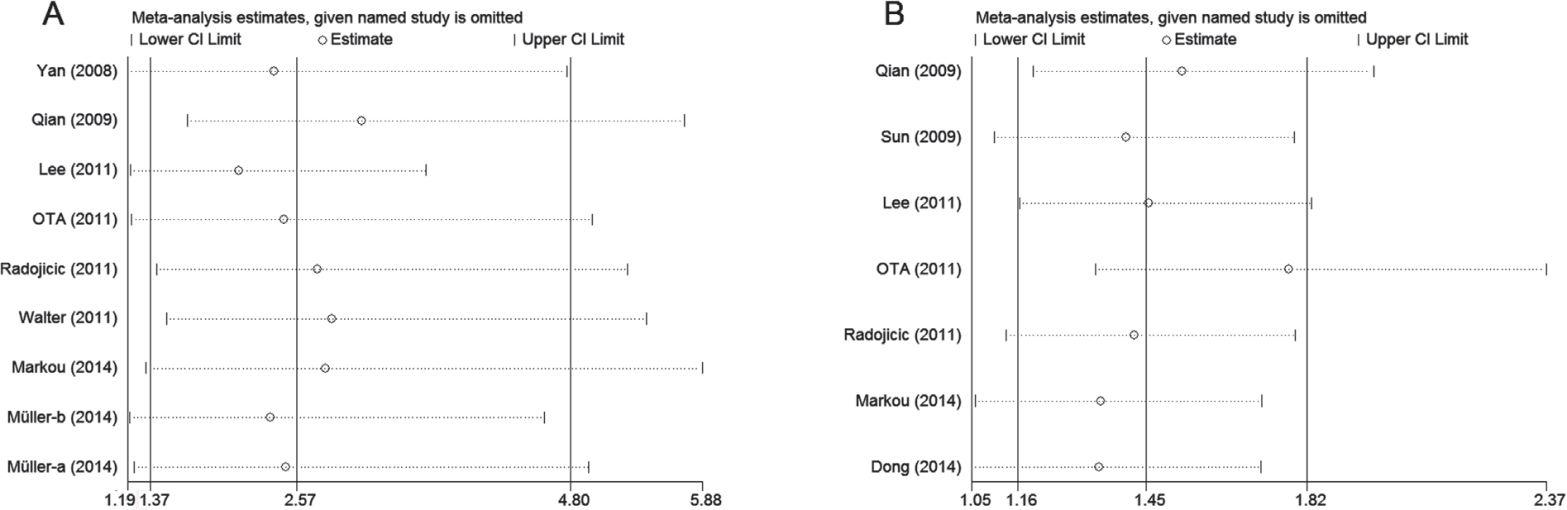
Sensitivity analysis for OS (A) and DFS/RFS (B).

Finally, the publication bias of the included studies was evaluated through funnel plots and Egger's tests. As shown in Fig. 3, the funnel plots were almost symmetric in OS studies, as well as in DFS/RFS studies. The corresponding *P* values of the Egger's regression intercepts were 0.859 and 0.312, thereby indicating that the meta-analysis did not display publication bias. Meanwhile, one study was omitted to measure its effects on the pooled HR for the OS or DFS/RFS in the sensitivity analysis. No individual study dominantly influenced overall HR, as presented in Fig. 4.

## Discussion

As mentioned previously, miR-21 is among the most significantly up-regulated miRNAs in many human cancers, including breast, colon, lung, prostate, ovarian, and stomach cancers [13,45]. The biological characteristics of miR-21 may explain the relationship between its expression and cancer outcome. The miR-21 gene is located in chromosome 17q23.2 within the common fragile site FRA17B. It is often amplified in numerous malignancies. MiR-21 can promote tumor development by down-regulating several tumor suppressor genes. Its identified direct targets include programmed cell death 4, tropomyosin 1, reversion-inducing cysteine-rich protein with kazal, and mapsin [14]. Si *et al*. showed that suppression or knock-down miR-21 in breast cancer MCF-7 cells can inhibit cell growth and induce apoptosis by downregulating the anti-apoptotic Bcl-2 [46].

Recently, a series of quantitative analyses have been carried out to identify the prognostic role of miR-21 in various cancers. Fu *et al*. demonstrated that high-level miR-21 predicts unfavorable overall survival in general carcinomas (HR = 1.69, 95% CI: 1.33–2.16, *P* < 0.001), especially head and neck squamous cell and digestive system carcinomas, moderately well on the basis of 17 studies [47]. Similar results were obtained in Zhou and Zhu’s reports, with pooled HR for OS 1.91 (95%CI: 1.66–2.19, *P* < 0.001) and 1.903 (95% CI: 1.713–2.113, *P* < 0.001), respectively [48,49]. In colorectal cancer, Xia *et al*. reported that higher miR-21 expression also indicates poorer survival with the combined HR to be 1.76 (95% CI: 1.34–2.32, *P* < 0.001) for OS [50]. However, insignificant or opposite results were also observed in some studies. In a meta-analysis of 1,163 non-small cell lung cancer (NSCLC) cases, Ma *et al*. showed the HR for OS is 2.19 (95% CI: 0.76–6.30, *P* = 0.15), which implies the miR-21 expression has limited prognostic significance on NSCLC in spite of the relationship between miR-21 expression and DFS/RFS (HR = 2.31, 95% CI: 1.52–3.49, *P* < 0.001) [51]. Overall, controversy concerning the prognostic role of miR-21 in cancers still exists. Only one study pooled the HR for OS in breast cancer and the dominant across-study heterogeneity, as well as small number of studies, made their results comparatively weak [48]. Here we performed a meta-analysis including 10 articles to comprehensively evaluate the risk of elevated miR-21 for survival in breast cancer patients.

Elevated miR-21 is correlated with poor survival rates (both OS and DFS) in breast cancer patients in this comprehensive meta-analysis of 1,439 cases from 10 cohorts. Nevertheless, the analysis results of DFS/RFS studies must be considered because they are only weakly significant based on their HR value of 1.45. A prognostic factor with RR > 2 is presumably useful in practice as per Hayes [52]. In addition, the results of the ethnicity subgroup analysis indicated that increased miR-21 expression significantly predicted low OS in Asians (HR = 5.07, 95% CI: 2.89–8.92, *P <* 0.001), whereas the prediction was not statistically significant in Caucasians (HR = 1.44, 95% CI: 0.99–2.10, *P* = 0.058). MiRNAs display different expression levels and predictive values across various ethnic groups [51,53]. In this sense, miR-21 is a suitable biomarker for breast cancer prognosis in Asians. Heterogeneity was not eliminated in the mean group during the cut-off subgroup analysis of OS. Moreover, only two studies used mean as the cut-off value and they adopted inconsistent mean values. Therefore, it was not appropriate to conclude that the up-regulated miR-21 significantly associated breast cancer prognosis in the mean cut-off group despite HR >2. As we all know, breast cancer is a kind of heterogeneous disease and its prognosis is closely determined by biological subtypes [54]. Hence, we performed subgroup analysis stratified by types of breast cancer. Positive result was obtained in mixed as well as Her2+ type of breast cancer patients. However, due to limited number of triple-negative and Her2+ studies, the results must be interpreted with caution.

ORs for TNM stage and histological grade were statistically significant in the correlation study of miR-21 expression on the clinical characteristics of patients, whereas the OR of lymph node metastasis was insignificant. This negative result is reasonable given the small population of each study and the diverse enrollment criteria although previous studies reported a connection between miR-21 and lymphoid infiltration in other cancers [51,55]. Furthermore, the process of metastasis was regulated by complicated molecular networks and not miR-21 alone. Currently, hormone receptors and Her2 status are important in the classification and management of breast cancer. The prognoses for Her2+ and triple-negative (Her2-/ER-/PR-) breast cancer types are usually poor because of the unique biology of the tumor itself and the lack of targeting therapy [4,56]. Our data indicated that miR-21 is positively associated with Her2 status but is negatively correlated to hormone receptors. This finding explains the low survival outcome of breast cancer patients with high miR-21 expression to some extent. However, it is noteworthy that the development of breast cancer can be influenced by the composition of tumor subtypes. In our meta-analysis, all studies for ORs calculation did not set limitations on the subtypes except Dong’s research. But no eminent difference was observed if we removed Dong’s study (Data not shown), which suggested the results were not remarkably affected by the type of breast cancer. Of importance, the association between clinical parameters and miR-21 expression need further investigation due to small number of studies and the sample size.

Although the meta-analysis determined the predictive effect of miR-21, especially in the Asian group, this study is limited in several ways. First, the marked heterogeneity of the subjects in the OS group is caused by the differences in the baseline characteristics of patients (age, tumor stage, race, or country), sample sites, the cut-off values of miR-21, the follow-up durations, and treatment strategies. A random-effect model was used in an attempt to minimize the effect of these differences because they may confound the study results. Simultaneously, we conducted subgroup analyses according to ethnic background, miR-21 cut-off value and types of breast cancer. Heterogeneity diminished significantly in the ethnicity subgroup but not in cut-off and type subgroup. These factors should be scrutinized when the conclusions concerned are considered. Second, we calculated several HRs based on data extracted from the survival curve; thus, these values may be slightly erroneous. Meanwhile, three studies reported only the unadjusted HR estimate for survival outcomes [38,42,43]; as a result, HR may have been overestimated. Third, the number of studies regarding specifically miR-21 and the prognosis of breast cancer was small, especially for ORs calculation and subgroup analysis, which was relatively insufficient to confirm the conclusion although no significant publication bias was detected in the meta-analysis. Moreover, we should carefully consider the disregard of other language articles and the preference for publications with significant results. Finally, we failed to specifically define miR-21 over-expression given the lack of uniform cut-off value although most studies defined the median as the cut-off of elevated miR-21 expression.

Furthermore, several concerns should be addressed to practically apply the prognostic value of miR-21. First, only one study detected the miR-21 expressions in serum, while others used tumor tissue or bone marrow samples. As demonstrated in other studies [57], a simultaneous detection of miRNA in serum may conveniently provide additional information regarding host response and prognosis. Hence, researchers should investigate the prognostic value of miR-21 circulation in breast cancer. Second, the lack of a golden standard for the miR-21 cut-off value complicated the exploration of its clinical application. Therefore, the cut-off value of miR-21 level should be clearly defined based on the global population. Third, the examination of a panel of miRNAs may yield more sensitive and specific results than that of a single miRNA given the complex oncogenesis process. For example, Liu *et al*. showed that a five-miRNA signature (miR-1, miR-20a, miR-27a, miR-34, and miR-423-5p) can serve as a better biomarker for gastric cancer screening than either a carcinoembryonic antigen or a carbohydrate antigen19-9 alone [58].

In conclusion, the present meta-analysis provided statistical evidence that miR-21 up-regulation can predict unfavorable breast cancer prognosis, especially in Asians. Our data also indicated a correlation between miR-21 expression and clinical parameters. Nonetheless, large and well-designed prospective studies should be conducted to confirm these findings before miR-21 can be implemented into routine clinical management.

## Supporting Information

S1 PRISMA Checklist (DOC)

S2 MOOSE Checklist (DOC)

S1 Table MiR-21 expression in respect to patients’ clinicopathological characteristics. (DOC)
