# Supplementary material for: Prediction of Poor Prognosis in Breast Cancer Patients Based on MicroRNA-21 Expression: A Meta-Analysis
Source: PLoS One. 2015 Feb 23;10(2):e0118647. doi: 10.1371/journal.pone.0118647 (PMC4338069; doi:10.1371/journal.pone.0118647)
Supplement: S2 MOOSE Checklist — (DOC) [file pone.0118647.s002.doc]

**Checklist S2. MOOSE Checklist**

| **Reporting of background should include** | |
| --- | --- |
| Problem definition | Introduction |
| Hypothesis statement | Introduction |
| Description of study outcome(s) | OS, DFS, RFS |
| Type of exposure or intervention used | Cancer |
| Type of study designs used | Meta-analysis |
| Study population | Global |
| **Reporting of search strategy should include** | |
| Qualifications of searchers (eg, librarians and investigators) | Stated in Materials and Methods |
| Search strategy, including time period included in the synthesis and keywords | Materials and Methods |
| Effort to include all available studies, including contact with authors | We contact authors and searched reference lists and citations |
| Databases and registries searched | Materials and Methods |
| Search software used, name and version, including special features used (eg, explosion) | Endnote X6 |
| Use of hand searching (eg, reference lists of obtained articles) | We hand-searched references of retrieved papers for additional references |
| List of citations located and those excluded, including justification | Flow diagram in Figure 1. |
| Method of addressing articles published in languages other than English | We included articles published in Chinese other than English |
| Method of handling abstracts and unpublished studies | We did not include unpublished or abstract only publications |
| Description of any contact with authors | When needed, we contacted the corresponding author for original data |
| **Reporting of methods should include** | |
| Description of relevance or appropriateness of studies assembled for assessing the hypothesis to be tested | Detailed inclusion and exclusion criteria were described in Materials and Methods section |
| Rationale for the selection and coding of data (eg, sound clinical principles or convenience) | Materials and Methods-Study selection/Data extraction |
| Documentation of how data were classified and coded (eg, multiple raters, blinding, and interrater reliability) | Results, Materials and Methods-Study selection/Data extraction/ Quality assessment |
| Assessment of confounding (eg, comparability of cases and controls in studies where appropriate) | We only included studies where appropriate controls were mentioned |
| Assessment of study quality, including blinding of quality assessors; stratification or regression on possible predictors of study results | Materials and Methods-Quality assessment |
| Assessment of heterogeneity | Materials and Methods-Statistical analysis |
| Description of statistical methods (eg, complete description of fixed or random effects models, justification of whether the chosen models account for predictors of study results, dose-response models, or cumulative meta-analysis) in sufficient detail to be replicated | Materials and Methods-Statistical analysis |
| Provision of appropriate tables and graphics | We included 1 flow diagram, 2 summary table, 1 forest plot of all studies, 1 subgroup analysis table, 1 odd ratio table and 1 sensitivity analyses figure. |
| **Reporting of results should include** | |
| Graphic summarizing individual study estimates and overall estimate | Figure 2 |
| Table giving descriptive information for each study included | Table 1, Table 2 |
| Results of sensitivity testing (eg, subgroup analysis) | Results, Table 3, Figure 4 |
| Indication of statistical uncertainty of findings | Discussion |
| **Reporting of discussion should include** | |
| Quantitative assessment of bias (eg, publication bias) | Results, Figure 3 |
| Justification for exclusion (eg, exclusion of non–English-language citations) | Discussion |
| Assessment of quality of included studies | See above |
| **Reporting of conclusions should include** | |
| Consideration of alternative explanations for observed results | Discussion |
| Generalisation of the conclusions (ie, appropriate for the data presented and within the domain of the literature review) | Discussion |
| Guidelines for future research | Discussion |
| Disclosure of funding source | Submission system |
