# Supplementary material for: Prediction of Poor Prognosis in Breast Cancer Patients Based on MicroRNA-21 Expression: A Meta-Analysis
Source: PLoS One. 2015 Feb 23;10(2):e0118647. doi: 10.1371/journal.pone.0118647 (PMC4338069; doi:10.1371/journal.pone.0118647)
Supplement: S1 Table — (DOC) [file pone.0118647.s003.doc]

**Table S1.** MiR-21 expression in respect to patients clinicopathological characteristics

| Study | Year | High miR-21 | | Low miR-21 | |
| --- | --- | --- | --- | --- | --- |
|  |  | Lymph node metastasis | Total | Lymph node metastasis | Total |
| Yan | 2008 | 19 | 61 | 30 | 52 |
| OTA | 2011 | 25 | 47 | 92 | 244 |
| Lee | 2011 | 17 | 31 | 30 | 78 |
| Walter | 2011 | 10 | 11 | 6 | 12 |
| Dong | 2014 | 22 | 38 | 13 | 34 |
|  |  | Histological grade (III) | Total | Histological grade (III) | Total |
| OTA | 2011 | 21 | 47 | 53 | 244 |
| Lee | 2011 | 18 | 31 | 24 | 78 |
| Walter | 2011 | 9 | 13 | 8 | 12 |
| Dong | 2014 | 27 | 38 | 12 | 34 |
|  |  | Her2 positive | Total | Her2 positive | Total |
| Yan | 2008 | 11 | 61 | 6 | 52 |
| OTA | 2011 | 15 | 37 | 17 | 133 |
| Lee | 2011 | 19 | 31 | 22 | 77 |
|  |  | PR positive | Total | PR positive | Total |
| Yan | 2008 | 33 | 61 | 32 | 52 |
| OTA | 2011 | 17 | 46 | 149 | 240 |
| Lee | 2011 | 14 | 31 | 51 | 78 |
| Walter | 2011 | 3 | 8 | 3 | 8 |
|  |  | ER positive | Total | ER positive | Total |
| Yan | 2008 | 30 | 61 | 27 | 52 |
| OTA | 2011 | 24 | 47 | 170 | 244 |
| Lee | 2011 | 14 | 31 | 59 | 78 |
| Walter | 2011 | 2 | 8 | 1 | 8 |
|  |  | TNM stage (III/IV) | Total | TNM stage (III/IV) | Total |
| Yan | 2008 | 19 | 61 | 5 | 52 |
| Lee | 2011 | 9 | 31 | 13 | 78 |
| Dong | 2014 | 27 | 38 | 12 | 34 |
